# Mr. Clement, on Herpetic Eruptions

**Published:** 1805-05-01

**Authors:** W. Clement

**Affiliations:** Shrewsbury


### *To the Editors of the Medical and Phyfical Journal.*

GENTLEMEN,

IN the numerous communications constantly inserted in your very useful Journal on Vaccination, I have been much surprised that no gentleman hath yet noticed those excellent Observations of Dr. Jenner on Herpetic Eruptions, published in your Journal of August last. Before I had read that valuable letter, I had long observed the often imperfect progress of the cow-pock pustule, in patients who had any irritative eruptive complaint. I not only found more difficulty in communicating the disease, but I had always been more or less dissatisfied with its influence on the system, as well as to external appearance. I am now convinced, that more real imperfect cases of vaccination hath occurred from this source, than any other; therefore medical gentlemen ought to be particularly cautious in minutely examining, before inoculation, whether their patients are free from all species of herpes, or itch; the latter, as well as the former, often impede the cow-pock pustule from proceeding regularly. Scrophula, on the contrary, seems to have no such effect. I have lately vaccinated a child, who had several large scrophulous ulcers, ers, yet passed thro' the disease properly and regularly; the wounds are now healed, and the child's general health appears much improved. Dr. Wood, in his History of Vaccination at Newcastle, in your last Journal, quotes Mr. Fife, respecting some slight eruptions about a child's ear; but he is totally silent on those sterling instructions, as well as observations, given us by Dr. Jenner, whom the world may still look up to for further information on this interesting subject. I can add but little weight to such authority. I shall only select one case, among many others, which I had a fair opportunity of witnessing. Mr. F's two children were inoculated with fresh vaccine fluid, on the 5th of October last; the youngest, ten months old, a fine healthy child, went through the disease regularly; the eldest, two years and a half, had been subject to a violent herpetic eruption, since he was four months old. His arm on the second day appeared much inflamed; on the third and fourth, the inflammation was so vivid, that I pronounced the pustule to be irregular; from those days to the twelfth every appearance, both as to efflorescence and scabbing, were incomplete. The child continued for a month pretty free from eruptions, during which time I again attempted to vaccinate him; but he became so cross and turbulent that I was obliged to desist. I conceive this child certainly liable to variolous infection at any future period, unless he is more perfectly vaccinated.* The above case strikingly confirms the effect of irritative eruptions, in preventing perfect vaccination; both children were inoculated at the same time, with the same fluid; one had herpes, the other none; one passed the disease regularly, the other did not.

With begging medical men to pay more particular attention to this subject,

I am, &c.

W. CLEMENT.

*Shrewsbury, March* 12, 1805.

---

* What were the causes that frequently prevented variolous infection from taking place after repeated inoculations? Were they eruptions or some constitutional idiosyncrasy? Were those cases in Fullwood's Rents, and some others we have heard of, free from all cuticular eruption during vaccination?

